# Exploratory Analysis of ELP1 Expression in Whole Blood From Patients With Familial Dysautonomia

**DOI:** 10.1002/acn3.70254

**Published:** 2025-12-12

**Authors:** Alejandra González‐Duarte, Lucy Norcliffe‐Kaufmann, Maria Luisa Cotrina, Zenith Khan, Kaia Dalamo, Patricio Millar Vernetti, Matthew Lawless, Elisabetta Morini, Monica Salani, Marla Weetall, Jana Narasimhan, Agostino G. Rocha, Susan A. Slaugenhaupt, Horacio Kaufmann

**Affiliations:** ^1^ Department of Neurology New York University Grossman School of Medicine New York New York USA; ^2^ Syneos Health Princeton New Jersey USA; ^3^ Department of Neurology Massachusetts General Hospital and Harvard Medical School Boston Massachusetts USA; ^4^ Center for Genomic Medicine Massachusetts General Hospital Boston Massachusetts USA; ^5^ PTC Therapeutics Warren New Jersey USA

**Keywords:** ELP1 protein assay, familial dysautonomia, HSAN‐III, neurologic biomarker

## Abstract

**Background:**

Familial dysautonomia (FD) is a hereditary neurodevelopmental disorder caused by aberrant splicing of the *ELP1* gene, leading to a tissue‐specific reduction in ELP1 protein expression. Preclinical models indicate that increasing ELP1 levels can mitigate disease manifestations. A blood‐based ELP‐1 protein assay may provide a reliable way to monitor gene target engagement.

**Design and Methods:**

Using a newly developed radioimmunoassay, we quantified ELP1 protein levels in peripheral blood samples collected from 59 homozygous FD patients carrying the IVS20 + 6T>C mutation and 66 heterozygous carriers. To assess the reproducibility of the measurement, replicate samples were collected in 43 participants. Longitudinal variability was evaluated in 22 participants who underwent repeat sampling 1 year later.

**Results:**

ELP1 protein levels were significantly lower in FD patients compared to heterozygous carriers (244 ± 75 vs. 2210 ± 1031 pg/mL, *p* < 0.001). Replicate analysis of 43 paired samples showed strong consistency in ELP1 levels (*p* < 0.000). Repeat measurements 1 year after baseline showed longitudinal stability (*R* ^2^ = 0.827, *p* < 0.001). An ELP1 threshold of 492 pg/mL yielded a sensitivity of 80.2% (CI of 70.6 to 87.2%) and a specificity of 98.2% (95% CI of 90%–99%) with a positive likelihood ratio of 46.5, indicating that individuals with FD were over 46 times more likely to have ELP1 levels below this threshold compared to non‐affected carriers.

**Conclusion:**

Blood ELP1 levels are robust and reproducible, with concentrations below 492 pg/mL strongly indicative of disease. Moreover, given their longitudinal stability, ELP1 can serve as a marker of target engagement to evaluate the efficacy of gene‐targeted therapies aimed at correcting ELP1 gene splicing and protein production.

## 1 Background

Familial dysautonomia (FD, OMIM: 223900) is a rare autosomal recessive neurodevelopmental disorder characterized by sensory and autonomic dysfunction [1, 2, 3]. Nearly all patients are homozygous for the IVS20 + 6T>C founder mutation in the *ELP1* gene, which causes a tissue‐specific splicing defect, leading to a reduction of ELP1 protein levels, particularly in neurons [2, 3]. ELP1 is a core component of the Elongator complex, which is required for transcriptional elongation, tRNA modification, and cytoskeletal organization [4, 5]. ELP1 deficiency disrupts neuronal development and survival leading to the multisystem phenotype of FD [6, 7, 8, 9, 10].

Preclinical studies in FD mouse models showed that even modest restoration of ELP1 via gene replacement or splicing modulator compounds rescues key disease features including retinal degeneration and gait ataxia [11, 12, 13, 14]. These findings support therapeutic approaches currently under development including splicing modulator compounds, antisense oligonucleotides, gene therapy, modified exon‐specific U1 small nuclear RNAs, and engineered CRISPR‐Cas9 base editors [12, 14, 15, 16, 17, 18, 19, 20]. The beneficial effects of higher ELP1 levels in these studies appear to be dose‐dependent [14, 19]. However, the exact threshold level of ELP1 necessary for normal function and neuronal survival is unknown.

Clinical translation has been hampered by the absence of a sensitive and easily accessible biomarker of target engagement. Previous biomarker research has focused on quantifying mis‐spliced ELP1 gene transcripts or the ratio of mis‐spliced to wild type mRNA which are difficult to quantify and do not measure functional protein [19, 21]. A blood‐based ELP1 protein assay represents a more practical alternative, enabling direct, reproducible quantification from easily obtained blood samples.

In this manuscript, we describe the development and validation of a novel assay for quantifying ELP1 protein levels in blood. We evaluate its reproducibility across replicate and longitudinal samples and the threshold below which ELP1 deficiency distinguishes FD patients from heterozygous carriers.

## 2 Methods

### 2.1 Study Design

This was a cross‐sectional study nested within the New York University Familial Dysautonomia Cohort, the largest international observational study of genetically confirmed patients with FD followed prospectively from birth in the US, Israel, Europe, Australia, and Central and South America.

Patients and carriers were enrolled between December 2017 and January 2022. Inclusion criteria for FD patients were: (1) homozygosity for the IVS20 + 6T>C mutation; (2) age older than 2 years; and (3) clinical features of familial dysautonomia (including reduced deep tendon reflexes, blood pressure instability, and reduced pain/temperature sensation occurring from birth). Entry criteria for the heterozygous carriers included: (1) confirmation as heterozygous carriers of the IVS20 + 6T>C mutation; (2) age > 18 years; and (3) no history of severe neurological or cardiovascular disease. Most carriers were parents of affected patients. The study was approved by the Institutional Review Board of New York University, and all participants provided written consent/assent to participate.

### 2.2 Protocol

**FIGURE 1:**
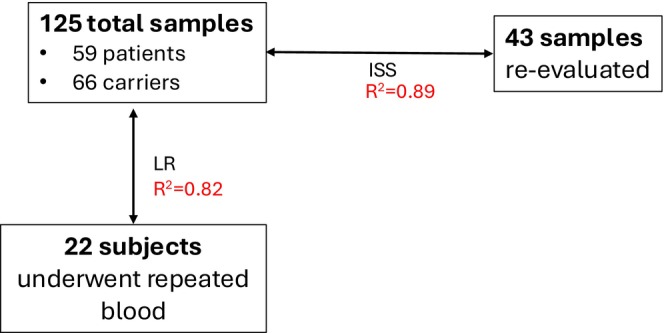
125total subjects. 43 subjects had replicate samples drawn, and 22 subjects had a second sample drawn after 1 year. ISS, incurred sample stability; LR, longitudinal repeat.

All subjects underwent blood collection through an antecubital vein. Samples were collected in replicate in 43 subjects to assess reproducibility. An additional 22 participants underwent repeat blood collection 1 year later to evaluate the test–retest reliability of these measures over time in an untreated cohort (Figure 1). Since there is currently no disease‐altering treatment for FD, this cohort provided a stable population to assess the consistency and reliability of the assay.

### 2.3 Sample Handling

Peripheral venous blood (10 mL) was collected in K2EDTA tubes, barcoded and placed in a −80°C freezer within 15 min. Samples were batched and stored locally until shipped overnight on dry ice to Syneos Health (301D College RD E Princeton, NJ, 08540) in a blinded fashion for analysis.

### 2.4 ELP1 Assay Development

An electrochemiluminescence (ECL) immunoassay was developed and validated by Syneos Health using the Meso Scale Discovery (MSD) platform. A mouse anti‐ELP1 capture antibody immobilized on MSD plates bound endogenous ELP1 from whole blood lysates. Detection was achieved with a rabbit anti‐ELP1 antibody followed by a sulfo‐tagged anti‐rabbit secondary antibody, generating an ECL signal proportional to ELP1 concentration. Quantitation was performed against a recombinant ELP1 standard curve (100–3200 pg/mL) using a 4‐parameter logistic regression with 1/y^2^ weighting using the GxP SoftMax Pro 7.0.3 analysis software. The assay was validated for precision, accuracy, selectivity, and stability; inter‐run precision was ≤ 7.6% and accuracy ranged from −1.6% to +3.3% across the calibration range. Stability studies demonstrated no loss of signal at 202 and 312 days of storage, with relative error within ±20% of baseline values. Screening of healthy donor samples during assay validation showed a broad physiologic range of ELP1 concentrations (≈1300–111,000 pg/mL). These findings confirm the assay's sensitivity, specificity, and reproducibility for quantifying ELP1 in patient samples, and meet the criteria described by the ICH‐M10 guidelines for a validated method [22].

### 2.5 Statistical Analysis

Analysis included all participants with baseline ELP‐1 samples that passed quality control. Descriptive statistics were generated for baseline characteristics, expressed as mean ± SD, or median and IQR for continuous variables and frequency/percentages for categorical variables.

Group comparisons were performed using the Chi‐square test for categorical variables. For continuous variables, independent *t*‐tests were applied when data were normally distributed. Otherwise, the Mann–Whitney *U* test was used. Analysis was performed with IBM SPSS Statistics Software (version 29.0, 2023), and Prism Software was used for data visualization (Version 10 (GraphPad Software Inc)). All *p* values were two‐tailed, and *p* < 0.05 was considered statistically significant.

### 2.6 IRB Approval

This study was approved by the Institutional Review Board (IRB) at NYU Grossman School of Medicine under IRB protocol number 116‐1774. Informed consent was obtained from all participants or their legal guardians prior to enrollment.

## 3 Results

### 3.1 Subject Characteristics

After quality control procedures, ELP‐1 protein quantification was successfully completed in 125 subjects, including 59 homozygous individuals with FD and 66 heterozygous carriers who served as controls. Table 1 shows the demographic and clinical characteristics of the participants. As expected, the FD group was significantly younger than the heterozygous carriers who were mostly parents (mean age: 23.4 years, range 4–62 years, vs. 52.4 years, range 38–71 years; *p* < 0.000). Given the age disparity, age was used as a covariate in all subsequent comparative analyses.

**TABLE 1:** Demographic characteristics.

	Patients (*n* = 59)	Controls (*n* = 66)
Age (years)	23.4 ± 11.5	52.4 ± 10.3
Gender		
Males	31 (53%)	24 (36%)
Females	28 (47%)	42 (64%)
ELP1 levels (pg/mL)		
All	278 ± 82.8	2221 ± 691.9
Males	304 ± 89.4	2166 ± 753.0
Females	251 ± 65.9	2254 ± 659.3
< 20 years	328 ± 99.1	NA
21–30 years	270 ± 87.2	1930 ± 658.0
31–40 years	307 ± 90.0	2084 ± 605.5
41–60 years	386 ± 23.6	2186 ± 813.6
> 61 years	NA	2360 ± 337.9
Repeated ELP1 levels (pg/mL)		
ELP1 levels (test)	244 ± 75.8	2210 ± 1031
ELP1 levels (retest)	270 ± 127	1985 ± 913

### 3.2 ELP1 Protein Levels

On average, ELP1 protein levels in whole blood were significantly lower in homozygous FD participants than in heterozygous carriers (244 ± 75 vs. 2210 ± 1031 pg/mL, *p* < 0.001). Within the FD group, ELP1 levels did not differ significantly by sex (males: 267 ± 74.5 vs. FD females: 216 ± 77.4 pg/mL, *p* = NS). In contrast, among heterozygous carriers, females had significantly higher levels of ELP1 than males (2146 ± 654 vs. 1790 ± 914 pg/mL, *p* = 0.001). There was no correlation between age and ELP1 in either group. Despite interindividual variability, the affected child consistently demonstrated lower ELP1 levels than one or both parents.

### 3.3 Stability of ELP1 Measurements

**FIGURE 2:**
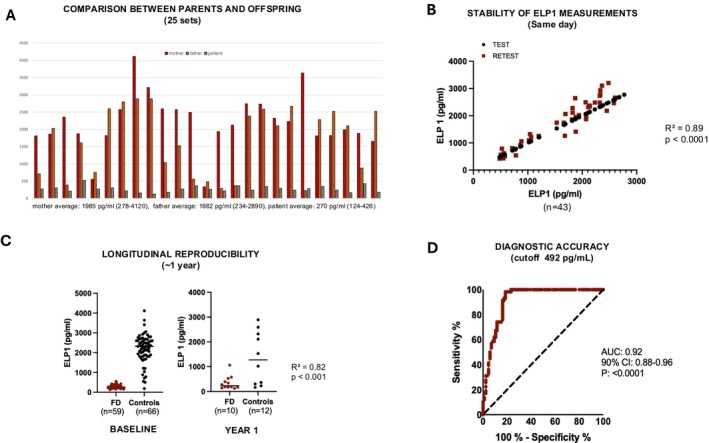
(A) Comparison of blood ELP1 protein levels between mother, father, and the affected child. Results showed that despite interindividual variability, the patient consistently had lower levels relative to one or both parents. (B) ELP1 protein levels measured in whole blood samples from FD patients and heterozygous controls in two independent tests. Correlation of ELP1 measurements between Test 1 and Test 2 demonstrated high reproducibility (*R* ^2^ = 0.89, *p* < 0.001), indicating assay consistency across runs. (C) ELP1 protein levels measured in whole blood samples from FD patients and heterozygous controls in two independent tests 1 year apart. ELP1 levels were significantly lower in FD patients compared to controls in both tests. (D) Receiver Operating Characteristic (ROC) curve illustrating the diagnostic performance of ELP1 levels for FD. The area under the curve (AUC) was 0.92 (90% CI: 0.88–0.96, *p* < 0.0001), indicating excellent discrimination between FD patients and controls. At an optimal threshold of 492.5 pg/mL, the assay achieved a sensitivity of 80.2% and specificity of 98.2%.

Incurred Sample Stability (ISS) serves as a key validation step to demonstrate that ELP1 concentrations remain stable and reproducible under study conditions, ensuring the robustness of bioanalytical data. To this end, we analyzed 43 test–retest pairs of blood samples collected simultaneously and processed in parallel, enabling a direct comparison of repeated measures from identical specimens. Pearson correlation analysis showed a strong positive association between test and retest values (*R* ^2^ = 0.8966, 95% CI: 0.9035–0.9711, *p* < 0.0001), indicating high reproducibility. These results confirm that analyte concentrations were consistent across paired samples, with minimal variability attributable to sample handling or assay performance (Figure 2A).

### 3.4 Reproducibility of ELP1 Measurements

To further evaluate biomarker reliability, we examined whether ELP1 concentrations remained consistent over longer time intervals. Longitudinal reproducibility was assessed in a subset of 22 subjects who underwent a second blood draw approximately 1 year after baseline. This analysis allowed us to determine whether intraindividual variability over time could affect the stability of the measure. ELP1 concentrations remained stable across the two time points, showing minimal within‐subject fluctuation. Correlation analysis demonstrated a strong positive relationship between baseline and follow‐up measurements (*R* ^2^ = 0.827, *p* < 0.001), confirming that ELP1 values are highly reproducible even over a 1‐year interval (Figure 2B).

### 3.5 Diagnostic Performance of ELP1 Thresholds

Finally, to define the optimal ELP1 cutoff for discriminating homozygous FD patients from heterozygous carriers, we assessed diagnostic accuracy across a range of thresholds. The criterion for selection was maximization of Youden's Index, a standard metric that combines sensitivity and specificity into a single measure of test performance. The highest index was observed at a threshold of 492 pg/mL, yielding a Youden's Index of 0.77, consistent with excellent overall diagnostic accuracy. At this cutoff, the assay demonstrated a sensitivity of 80.2% (95% CI: 80%–96%) and a specificity of 98.2% (95% CI: 90.8%–99.9%). The corresponding positive likelihood ratio was 46.5, indicating that individuals with FD were over 46 times more likely to have ELP1 levels below this threshold compared to non‐affected carriers (Figure 2C).

## 4 Discussion

Our study shows that ELP1 protein levels in blood are markedly reduced in FD patients compared with heterozygous carriers. These findings parallel prior reports of reduced ELP1 mRNA expression in leukocytes [23], but extend them by showing that the protein itself can be quantified directly in whole blood.

Several important implications emerge. First, ELP1 protein measurement provides a practical surrogate biomarker: It replicates the leukocyte mRNA signal but avoids the complexity of RNA‐based assays, offering a simpler, more scalable method for clinical application. Second, the data support the concept of a biological threshold below which ELP1 deficiency results in disease manifestation, underscoring the biomarker's diagnostic utility. Third, both replicate and longitudinal analyses showed high reproducibility, confirming robustness across different time points. Finally, an empirically defined cutoff (~492 pg/mL) offers a potential target threshold for monitoring pharmacodynamic effects in trials of splicing modulator compounds, antisense oligonucleotides, and gene therapies.

Development of a blood‐based biomarker for FD is essential given the rapid development of gene‐targeted therapies for FD. Other candidate biomarkers, such as serum and fecal metabolites, have also been reported to differ significantly between FD patients and healthy individuals [24, 25]. However, metabolite levels can be influenced by confounding factors such as gastrostomy tube feeding, recurrent antibiotic use, and nutritional status, which may affect reproducibility. On the other hand, blood ELP1 protein levels provide a major advantage over traditional tests of nerve function by allowing the inclusion of younger or more severely affected patients who might otherwise be excluded due to their inability to subjectively report symptoms.

The cellular source of ELP1 detected in whole blood is most likely leukocytes, which actively transcribe and translate ELP1 [23]. Mature erythrocytes lack nuclei, and platelets contain only residual protein, making them unlikely contributors [26, 27]. Importantly, while blood ELP1 levels are robust and reproducible, it remains uncertain whether changes in blood levels fully reflect ELP1 levels in neurons or retina, where disease pathology is most pronounced.

This study has limitations. The sample size was modest, reflecting the rarity of the disease, and replication in larger, independent cohorts will be needed to validate these findings. In addition, although ELP1 concentrations are robust and reproducible, it remains uncertain whether increasing circulating levels will translate into reduced disease severity or slower nerve degeneration. Another limitation is that normal reference ranges in healthy controls have not yet been established, which prevents a solid comparison across all groups and underscores the need for future studies. Finally, ELP1 measured in peripheral whole blood most likely reflects intracellular protein levels within leukocytes, but this may not be representative of all tissues, particularly in the nervous system where ELP1 expression is dramatically reduced. Normal reference ranges in healthy controls remain to be established, and the relationship between higher circulating ELP1 levels and clinical outcomes must be determined as gene‐targeted therapies become available.

In summary, our findings establish blood ELP1 protein quantification as a practical and reproducible biomarker for FD. It holds great promise for monitoring target engagement in therapeutic trials, and could potentially be used in diagnostic assessment in the future. Future studies should define normative ranges, assess longitudinal dynamics in larger cohorts, and explore its value alongside multimodal biomarkers to strengthen clinical utility.

## Author Contributions

A.G.‐D. clinically evaluated the participants, interpreted results, performed the statistical analysis, and wrote the first draft of the manuscript. L.N.‐K. designed the study and edited the manuscript. S.A.S., M.S., and E.M. helped with assay development, design of the study, and edited the manuscript. M.L. developed the RIA. M.L.C., Z.K., and K.D. collected blood samples and handled sample processing and reconciliation. M.W., J.N., A.G.‐D., and A.G.R. were involved in the assay development. H.K. designed and oversaw the study, clinically evaluated the participants and edited the manuscript.

## Disclosure

The authors have nothing to report.

## Consent

All authors read and consent for its publication.

## Conflicts of Interest

The authors declare no conflicts of interest.

## Data Availability

The data that support the findings of this study are available on request from the corresponding author. The data are not publicly available due to privacy or ethical restrictions.

## References

[1] F. B. Axelrod , “Familial Dysautonomia,” Muscle & Nerve 29, no. 3 (2004): 352–363. 14981733 10.1002/mus.10499

[2] A. Gonzalez‐Duarte , M. Cotrina‐Vidal , H. Kaufmann , and L. Norcliffe‐Kaufmann , “Familial Dysautonomia,” Clinical Autonomic Research 33, no. 3 (2023): 269–280. 37204536 10.1007/s10286-023-00941-1

[3] L. Norcliffe‐Kaufmann , S. A. Slaugenhaupt , and H. Kaufmann , “Familial Dysautonomia: History, Genotype, Phenotype and Translational Research,” Progress in Neurobiology 152 (2017): 131–148. 27317387 10.1016/j.pneurobio.2016.06.003

[4] B. Huang , M. J. Johansson , and A. S. Bystrom , “An Early Step in Wobble Uridine tRNA Modification Requires the Elongator Complex,” RNA (New York, N.Y.) 11, no. 4 (2005): 424–436. 15769872 10.1261/rna.7247705 PMC1370732

[5] C. Creppe , L. Malinouskaya , M. L. Volvert , et al., “Elongator Controls the Migration and Differentiation of Cortical Neurons Through Acetylation of Alpha‐Tubulin,” Cell 136, no. 3 (2009): 551–564. 19185337 10.1016/j.cell.2008.11.043

[6] L. Norcliffe‐Kaufmann , F. Axelrod , and H. Kaufmann , “Afferent Baroreflex Failure in Familial Dysautonomia,” Neurology 75, no. 21 (2010): 1904–1911. 21098405 10.1212/WNL.0b013e3181feb283 PMC2995385

[7] L. Norcliffe‐Kaufmann , F. B. Axelrod , and H. Kaufmann , “Developmental Abnormalities, Blood Pressure Variability and Renal Disease in Riley Day Syndrome,” Journal of Human Hypertension 27, no. 1 (2013): 51–55. 22129610 10.1038/jhh.2011.107 PMC3318957

[8] L. Norcliffe‐Kaufmann and H. Kaufmann , “Familial Dysautonomia (Riley‐Day Syndrome): When Baroreceptor Feedback Fails,” Autonomic Neuroscience 172, no. 1–2 (2012): 26–30. 23178195 10.1016/j.autneu.2012.10.012

[9] C. E. Mendoza‐Santiesteban , T. R. Hedges, 3rd , L. Norcliffe‐Kaufmann , et al., “Clinical Neuro‐Ophthalmic Findings in Familial Dysautonomia,” Journal of Neuro‐Ophthalmology 32, no. 1 (2012): 23–26. 21918475 10.1097/WNO.0b013e318230feab PMC6022825

[10] C. E. Mendoza‐Santiesteban , I. T. R. Hedges , L. Norcliffe‐Kaufmann , F. Axelrod , and H. Kaufmann , “Selective Retinal Ganglion Cell Loss in Familial Dysautonomia,” Journal of Neurology 261, no. 4 (2014): 702–709. 24487827 10.1007/s00415-014-7258-2

[11] E. Morini , D. Gao , C. M. Montgomery , et al., “ELP1 Splicing Correction Reverses Proprioceptive Sensory Loss in Familial Dysautonomia,” American Journal of Human Genetics 104, no. 4 (2019): 638–650. 30905397 10.1016/j.ajhg.2019.02.009 PMC6451698

[12] M. Ajiro , T. Awaya , Y. J. Kim , et al., “Therapeutic Manipulation of IKBKAP Mis‐Splicing With a Small Molecule to Cure Familial Dysautonomia,” Nature Communications 12, no. 1 (2021): 4507. 10.1038/s41467-021-24705-5 PMC8302731 34301951

[13] A. Schultz , S. Y. Cheng , E. Kirchner , et al., “Reduction of Retinal Ganglion Cell Death in Mouse Models of Familial Dysautonomia Using AAV‐Mediated Gene Therapy and Splicing Modulators,” Scientific Reports 13, no. 1 (2023): 18600. 37903840 10.1038/s41598-023-45376-w PMC10616160

[14] E. Morini , A. Chekuri , E. M. Logan , et al., “Development of an Oral Treatment That Rescues Gait Ataxia and Retinal Degeneration in a Phenotypic Mouse Model of Familial Dysautonomia,” American Journal of Human Genetics 110, no. 3 (2023): 531–547. 36809767 10.1016/j.ajhg.2023.01.019 PMC10027479

[15] S. Yun , A. Chekuri , J. Art , et al., “Engineered CRISPR‐Base Editors as a Permanent Treatment for Familial Dysautonomia,” *bioRxiv*, (2024), 10.1101/2024.11.27.625322.

[16] R. Sinha , Y. J. Kim , T. Nomakuchi , et al., “Antisense Oligonucleotides Correct the Familial Dysautonomia Splicing Defect in IKBKAP Transgenic Mice,” Nucleic Acids Research 46, no. 10 (2018): 4833–4844. 29672717 10.1093/nar/gky249 PMC6007753

[17] S. T. Hatch , A. A. Smargon , and G. W. Yeo , “Engineered U1 snRNAs to Modulate Alternatively Spliced Exons,” Methods 205 (2022): 140–148. 35764245 10.1016/j.ymeth.2022.06.008 PMC11185844

[18] G. Romano , F. Riccardi , E. Bussani , et al., “Rescue of a Familial Dysautonomia Mouse Model by AAV9‐Exon‐Specific U1 snRNA,” American Journal of Human Genetics 109, no. 8 (2022): 1534–1548. 35905737 10.1016/j.ajhg.2022.07.004 PMC9388384

[19] I. Donadon , M. Pinotti , K. Rajkowska , et al., “Exon‐Specific U1 snRNAs Improve ELP1 Exon 20 Definition and Rescue ELP1 Protein Expression in a Familial Dysautonomia Mouse Model,” Human Molecular Genetics 27, no. 14 (2018): 2466–2476. 29701768 10.1093/hmg/ddy151 PMC6030917

[20] A. Chekuri , K. Kondabolu , E. G. Kirchner , et al., “AAV2‐Mediated Intravitreal Delivery of Exon‐Specific U1 snRNA Rescues Optic Neuropathy in a Mouse Model of Familial Dysautonomia,” *bioRxiv*, (2025), 10.1101/2025.08.21.671454. PMC12797049 41536810

[21] D. Gao , E. Morini , M. Salani , et al., “A Deep Learning Approach to Identify Gene Targets of a Therapeutic for Human Splicing Disorders,” Nature Communications 12, no. 1 (2021): 3332. 10.1038/s41467-021-23663-2 PMC8185002 34099697

[22] U.S. Food and Drug Administration , M10 Bioanalytical Method Validation and Study Sample Analysis: Guidance for Industry (U.S. Food and Drug Administration, 2022), https://www.fda.gov/regulatory‐information/search‐fda‐guidance‐documents/m10‐bioanalytical‐method‐validation‐and‐study‐sample‐analysis.

[23] G. Gold‐von Simson , M. Leyne , J. Mull , et al., “IKBKAP mRNA in Peripheral Blood Leukocytes: A Molecular Marker of Gene Expression and Splicing in Familial Dysautonomia,” Pediatric Research 63, no. 2 (2008): 186–190. 18091349 10.1203/PDR.0b013e31815ef74b

[24] A. M. Cheney , S. M. Costello , N. V. Pinkham , et al., “Gut Microbiome Dysbiosis Drives Metabolic Dysfunction in Familial Dysautonomia,” Nature Communications 14, no. 1 (2023): 218. 10.1038/s41467-023-35787-8 PMC9839693 36639365

[25] S. M. Costello , A. M. Cheney , A. Waldum , et al., “A Comprehensive NMR Analysis of Serum and Fecal Metabolites in Familial Dysautonomia Patients Reveals Significant Metabolic Perturbations,” Metabolites 13, no. 3 (2023): 433. 36984872 10.3390/metabo13030433 PMC10057143

[26] S. D. Kumar , D. Kar , M. N. Akhtar , et al., “Evidence for Low‐Level Translation in Human Erythrocytes,” Molecular Biology of the Cell 33, no. 12 (2022): br21. 35976696 10.1091/mbc.E21-09-0437 PMC9635303

[27] E. Boilard , D. Burger , E. Buzas , et al., “Deciphering Platelets: Are They Cells or an Evolved Form of Extracellular Vesicles?,” Circulation Research 136, no. 4 (2025): 442–452. 39946441 10.1161/CIRCRESAHA.124.324721 PMC11839173

